# Hand hygiene compliance in Dutch general practice offices

**DOI:** 10.1186/s13690-020-00464-5

**Published:** 2020-09-12

**Authors:** Nataliya Hilt, Mariëtte Lokate, Alfons OldeLoohuis, Marlies E. J. L. Hulscher, Alex W. Friedrich, Andreas Voss

**Affiliations:** 1 Radboudumc, Department of Medical Microbiology, Geert Grooteplein Zuid 10, 6525 GA Nijmegen, The Netherlands; 2 Department of Medical Microbiology, University of Groningen, University Medical Center Groningen, Hanzeplein 1, 9713 GZ Groningen, The Netherlands; 3 Radboudumc, Department of Primary and Community Care, Geert Grooteplein Zuid 10, 6525 GA Nijmegen, the Netherlands; 4 Scientific Institute for Quality of Healthcare, Radboud University Nijmegen Medical Centre, Geert Grooteplein Zuid 10, 6525 GA Nijmegen, the Netherlands; 5 Department of Clinical Microbiology and Infectious Diseases, Canisius-Wilhelmina Hospital (CWZ), Weg door Jonkerbos 100, 6532 SZ Nijmegen, the Netherlands; 6 Radboudumc, REshape Center for Innovation, Geert Grooteplein Zuid 10, 6525 GA Nijmegen, the Netherlands

**Keywords:** Hand hygiene, General practitioners, Alcohol-based hand rub, Primary care

## Abstract

**Background:**

Hand hygiene (HH) is considered one of the most important measures to prevent healthcare-associated infections (HAI). Most studies focus on HH compliance within the hospital setting, whereas little is known for the outpatient setting. The aim of this study was to evaluate compliance with HH recommendations in general practitioners (GPs) office, based on World Health Organization (WHO) guideline.

**Methods:**

An observational study was conducted at five Dutch GPs-practices in September 2017. We measured HH compliance through direct observation using WHO’s ‘five moments of hand hygiene’ observation tool. All observations were done by one trained professional.

**Results:**

We monitored a total of 285 HH opportunities for 30 health care workers (HCWs). The overall compliance was 37%. Hand hygiene compliance was 34, 51 and 16% for general practitioners, practice assistants, and nurses, respectively. It varies between 63% after body fluid exposure and no HH performance before-, during and after home visit of a patient (defined as moment 5). The preferred method of HH was soap and water (63%) versus 37% for alcohol-based hand rub (ABHR). The median time of disinfecting hands was 8 s (range 6–11 s) for HCWs in our study.

**Conclusions:**

HH compliance among HCWs in Dutch GPs was found to be low, especially with regard to home visits. The WHO recommended switch from hand wash to ABHR was not implemented by the majority of HCWs in 5 observed GPs offices.

## Background

In 1847, Semmelweis has already reported about the importance of hand hygiene (HH) in the control of infection [1]. While hands certainly are a relevant route of transmission of infection, including outpatient care setting (for example, visit to a general practitioner’s (GPs) office) [2], the effectiveness of good hand hygiene has mainly been demonstrated in institutionalized healthcare [3–5]. Despite this evidence of hand contamination and colonization by potentially harmful microorganisms [2], very few reports of outbreaks in outpatient settings have identified hands as the transmission route of the causative microorganisms [6, 7].

The World Health Organization (WHO) recommendations on hand hygiene best practices and improvement strategies within its campaign ‘Clean Care is Safer Care’ [8] are considered the gold standard for health-care worldwide. Evaluation and feedback of HH performance are important elements of this program. Direct unobtrusive observation is recognized by WHO as the “gold standard” and most reliable method for measuring HH compliance rates [9]. Direct observation helps to pinpoint areas of strength or weaknesses in HH behavior, identify the number of HH opportunities, their indications, assess technique and provide feedback to healthcare workers (HCWs) [9, 10].

Numerous studies over the last few decades have shown that HH compliance is generally less than 50% of all the opportunities, with regard to both: hospital and outpatient setting [11–16]. Compliance can fluctuate depending on many different factors, such as type of healthcare provider (physician/nurse), or healthcare location (hospital/primary care). Adherence with HH guidelines in healthcare is considered a preventive behavior and should be approached as such [17]. Information on hand hygiene compliance and barriers for optimal hand hygiene practices at the general practitioners level remain limited [16, 18, 19]_._

The main aim of this study was to evaluate compliance with hand hygiene by health care workers in Dutch general practices according to the international recommendations of Hand Hygiene in Outpatient and Home-based Care and Long-term Care Facilities (a guide to the Application of the WHO Multimodal Hand Hygiene Improvement Strategy and the “My Five Moments for Hand Hygiene” Approach) [2] using the WHO Patient Safety Observation Form to document healthcare workers hand hygiene actions, based on the “My Five Moments for Hand Hygiene” of the World Health Organization [20].

The secondary aim was to identify potential factors that help (facilitators) or hinder (barriers) the performance of hand hygiene by these HCWs in daily practice.

## Methods

An observational study was conducted in five Dutch general practice offices in the Eindhoven region (located in the south of the Netherlands) in September, 2017.

Participants were classified as: all HCWs who are working at 5 general practice offices, i.e. 16 general practitioners (GPs) (including general practitioners in training), 9 practice assistants (PAs) and 5 nurse practitioners (NPs). PAs play a central role in the practice: they perform routine diagnostic and therapeutic interventions and serve as the patients’ point of contact for health education and the booking of practice visits. NPs in the Netherlands usually have a professional nursing background. NPs are qualified to diagnose medical problems, order treatments, perform non-surgical procedures and minor surgical procedures, and prescribe medications [21]. NPs in our study group performed chronic care management for patients with, for example, asthma, emphysema / chronic bronchitis and diabetes mellitus.

### Observation of hand hygiene compliance

According to the WHO strategy [20], compliance is defined as handwashing / -disinfection in an opportunity for hand hygiene. Hand hygiene opportunities were designated as appropriate or inappropriate per WHO criteria [20]. The five moments identified in this strategy [20] include (1) prior to patient contact, (2) prior to a clean or aseptic procedure, (3) after contact with body fluid, (4) after patient contact, and (5) after contact with the patient environment. Moment 5 is also included in the current national Infection Prevention (IP) guideline for GPs (which was issued by the Dutch Working Group on Infection Prevention (WIP) and Dutch GPs Society (NHG) in 2009 and recently up-dated) [22], but not defined. We defined moment 5 as HH performance before-, during and after home visit of a patient and not in a GP office.

The healthcare workers were observed during routine patient care visits. All observations were performed by one trained professional (NH), who was trained and then validated by the Department of Infection Prevention in an academic hospital according to recommendation of WHO Guidelines [20]. Validation took place by parallel observation jointly with a confirmed observer according to recommendation of WHO, namely: two observers engaged in an observation session during a real-life care situation and each completed an observation form separately, while observing the same HCW and the same care sequence. Results were then compared and discordant notifications discussed. This process was repeated until concordance was reached in the number and nature of each occurring hand hygiene opportunity [9].

To ensure quality of data, standardized checklists were used. The observer was present in the GP patient’s / examination room. Before the start of an observation moment, oral permission from each patient was received. In attempt to minimize the “Hawthorne effect” [23], none of the HCWs were aware of the fact that hand hygiene was the goal of observation. Instead, it was communicated that the purpose of observation was an infection-related subject without a concrete subject-matter. The majority of HCWs thought that the purpose of observation was the presence of IP protocols or choice of cleaning products, or the cleaning of nondisposable instruments etcetera. Looking back during interviews, nobody had thought that hand hygiene was the purpose of observation.

### Observation of hand hygiene duration and wrist and/or hand jewelry

Next to HH compliance, the duration of ABHR-procedure (using watch) and the wearing of wrist and/or hand jewelry, were measured during the observations.

### Professional and practice characteristics

After the observation period, to measure professional and practice characteristics, a questionnaire containing 7 items was used for each HCW (*N* = 30): age, gender, years of experience, form of GP practice, function of HCWs, training in IP and the preferred method of hand hygiene (soap and water or alcohol-based hand rub (ABHR)). The questionnaire on professional and practice characteristics also contained two open questions, one on facilitators and one on barriers to the performance of HH. All HCWs received the questionnaire after observation of HH had ended. After the study was completed, the results were shared with the all participants and practice manager. Suggestions for improvement were also provided.

### Analyses

Collected data was entered and analyzed using SPSS software version 22.0 (IBM). Descriptive statistics (frequencies) were applied.

## Results

### Study population

**Fig. 1:**
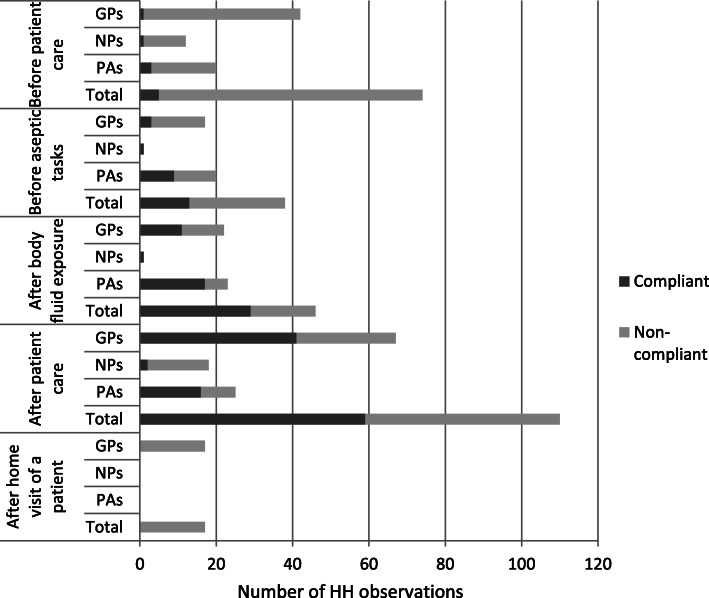
Hand hygiene (HH) performance of Health care workers: General practitioners (GPs), Nurse Practitioners (NPs) and Practice Assistants (PAs) in relation to ‘WHO 5 moments’

Thirty (30) HCWs were monitored; 16 general practitioners, 9 practice assistants and 5 nurse practitioners. The age of the healthcare providers ranged from 21 to 64 years (median 40.9 ± 11.4). Seventy-three percent of participants were female. For other baseline characteristics of the study population see Table 1. In total, 285 HH opportunities were observed (GPs 165, PAs 88 and NPs 32) (see Fig. 1). There were no outliers in HH performance between 5 GPs offices (data not shown).

**Table 1:** Demographic and basic characteristics of the Health care workers (*N* = 30)

		General Practioners	Nurse Practitioners	Practice Assistants
		Median (range)	Median (range)	Median (range)
Age (years)		36.5 (28–64)	38 (36–55)	40 (21–55)
Work experience (years)		6 (0.5–30)	6 (4–16)	14 (0.5–32)
		N (%)	N (%)	N (%)
Gender	Male	8 (50,0)	0	0
	Female	8 (50,0)	5 (100,0)	9 (100,0)
GPs offices forms	Solo	0	0	0
	Duo	7 (43,7)	2 (40,0)	3 (33,3)
	Group	9 (56,3)	3 (60,0)	6 (66,7)
HCWs trained in IP	Yes	4 (25,0)	0 (0,0)	3 (33,3)
	No	12 (75,0)	5 (100,0)	6 (66,7)
Carriage of Jewelry	Yes	10 (62,5)	4 (80,0)	5 (55,6)
	No	6 (37,5)	1 (20,0)	4 (44,4)

*GPs* General practitioners; *HCWs* Health care workers; *IP* Infection prevention

### Hand hygiene compliance

The overall HH compliance was 37% (i.e. performance of recommended HH in 106 from all 285 opportunities). HH compliance differed by profession: general practitioners, 34% (56/165); practice assistants, 51% (45/88), and nurse practitioners, 16% (5/32). Overall HH compliance with respect to the five moments varies between 63% after body fluid exposure and no HH performance before-, during and after home visit of a patient (defined as moment 5) (see Table 2). Overall HH compliance was 34% after glove use compared to 38% when gloves were not used. For HH compliance among GPs, PAs and NPs per moment see Fig. 1. According to the answers in the questionnaire, the preferred method of hand hygiene was soap and water (63%) versus 37% for alcohol-based hand rub (*N* = 30).

**Table 2:** Overall Hand hygiene compliance of health care workers: General practitioners (GPs), Nurse Practitioners (NPs) and Practice Assistants (PAs) in relation to ‘WHO 5 moments’

WHO 5 moments	Performance of recommended HH / opportunities, N	Compliance, %
Prior to patient care / to patient touching (moment 1)	5/74	6
Prior to a clean / aseptic procedure (moment 2)	13/38	34
After body fluid exposure (moment 3)	29/46	63
After contact with patients (moment 4)	59/110	54
Before-, during and after home visit of a patient (moment 5)	0/17	0

*WHO* The World Health Organization; *HH* Hand hygiene

### Duration of hand hygiene

The duration of hand disinfection was observed for 35 HH moments. Most observations were done for general practitioners (*N* = 23), followed by PAs (*N* = 8) and NPs (*N* = 4). The median time of disinfecting hands was 8 s (range 6–11 s). PAs disinfected their hands slightly longer than GPs and NPs (9 s versus 8 s).

### Wearing hand and wrist jewelry during patient care

The consensus recommendation (national and international) is to strongly discourage the wearing of rings or other jewelry during health care. So, we decided to observe the wearing of wrist and/or hand jewelry during the HH tasks. Sixty-three percent of HCWs wore hand and / or wrist jewelry during patient care (See Table 3).

**Table 3:** Number of health care workers, wearing hand and wrist jewelry during patient care

Health care workers, (N total)	Ring only, N	Watch only, N	Both Ring and Watch ± Bracelet, N	Totally with jewelry, N
General Practitioners, (16)	2	6	2	10
Practice Assistants, (9)	1	–	4	5
Nurse Practitioners, (5)	2	–	2	4

### Potential factors that help or hinder HH performance

About half of the HCWs mentioned barriers and facilitators in applying adequate HH practices. These barriers were: lack of intention (according to 8 HCWs); lack of knowledge about HH practice (according to 14 HCWs) including lack of knowledge about the indications and the 5 moments, and ignorance about proper technique of hand hygiene; the fear of side effects such as hand eczema when using ABHR (according to 12 HCWs); doubts about efficacy of HH procedure for non-hospitalized patients (according to 6 HCWs) and no infectious contact (5 HCWs). The facilitators were: interest in education about better HH practice (according to 12 HCWs); availability of pocket bottles with ABHR (according to 6 HCWs); placement of dispensers with ABHR along the walking route to the waiting room (according to 5 HCWs); placement of reminders in the workplace (according to 4 HCWs) and routine observation with feedback (3 HCWs).

## Discussion

Our study showed some prominent shortcomings in the general practice setting: low overall hand hygiene compliance. Before-, during and after home visits of a patient, even no hand hygiene was observed. The WHO recommended switch from hand wash to ABHR was not implemented by the majority of health care workers in the observed GP offices; 63% of HCWs wore hand and / or wrist jewelry during patient care and actual HH duration was significantly shorter than recommended.

Overall hand hygiene compliance of the study group was 37%. So far, most studies focus on HH compliance within the hospital setting. According to the systematic review of studies on compliance with HH guidelines in hospital care in industrialized countries, the authors found an overall median compliance rate of 40% among HCWs in 96 studies [24]. But some authors reported crucially lower HH compliance. Martin-Madrazo et al., for example, showed that the overall compliance rate with hand hygiene practice was 8,1% in observed primary healthcare in Madrid [16]. Similar to many studies in the literature, compliance of HH among nurses is better than doctors [25, 26]. In our study group, compliance (and number of opportunities) differed between the various professionals, with nurse practitioners scoring lower (16% (5/32)) than GPs (34% (56/165)). Practice assistants showed the highest performance scores (51% (45/88)).

The current study attempted to evaluate the performance of hand hygiene based on the WHO five moments. We showed that performance of HH varied between the various moments of hand hygiene; e.g. overall 6% before patient contact and 54% after patient contact.

The latter is not in line with our previous study about self-reported HH compliance of Dutch GPs, showing that 38 and 95% of GPs indicated to clean their hands before and after touching a patient, respectively [18]. This large difference between compliance by unobtrusive direct observation and self-reported compliance, concluded that practice is weakly correlated to self-reported behavior [27, 28] which in turn can depend on different determinants, such as lack of knowledge, risk perception, attitude, etcetera [29]. The fact that the HCWs were more likely to comply with hand hygiene after patient contact rather than before may reflect a priority to protect themselves from the patient’s body fluids rather than to protect the patient. This emphasizes the need for educational programs and increased surveillance to ensure both patients and HCWs are not being exposed to harmful organisms or transporting them to other areas.

According to our definition of 5th moment of HH, we observed no HH during the 17 opportunities (home visits) of 6 different GPs. We defined moment 5 as HH performance before-, during and after home visit of a patient and not in a GP office. No practical example of this moment of hand hygiene has been mentioned in Dutch IP guideline for GPs [22]. The WHO defines the 5th moment as hand hygiene being indicated after touching patient surroundings, i.e. ‘after touching any object or furniture when leaving the patient surroundings, without having touched the patient’ [9]. GPs in the Netherlands visit some of their patients at home. Dutch GPs are selective when deciding on a home visit: usually it is a very sick / old patient or sick child or it concerns a maternity visit. During such visits, minimal physical examination or aseptic action is often performed. There is, however, intensive contact with the patient’s environment, for example, touching the furniture (helping with bed settings to help a bedridden patient sit better in bed), or touching the chair that you sit on, the table you write on, but also the door that the GP grasps, etcetera. That is why we decided, to assess performance of HH moment 5, to observe HH during home visits to patients. The debate concerning the role of the environment in the cross-transmission of pathogens has been reignited [30, 31], suggesting that transmission of the causative microorganisms by this route is plausible. The fact that this moment of hygiene resulted in the lowest compliance level implies that future policies and research need to focus on the role of patient surroundings in cross-transmission. Also availability of pocket bottles with ABHR may contribute to better hand hygiene performance after contact with the patient environment.

Lastly, we belief that clear definition of the 5th moment of HH in Dutch IP guideline for GPs, with examples after contact with the patient environment, will help to improve HH performance.

Reasons which might explain suboptimal HH practices are multiple and may vary according to the setting and the resources available [32]. For example, perception and knowledge of the transmission risk, HCWs self-efficacy beliefs and the intention to perform HH [32, 33]. We saw the same barriers that help hinder HH performance (lack of knowledge and intention) in our top three hindering factors together with the fear of side effects such as hand eczema when using ABHR. This suggests that educational programs about skin health, indications and efficacy of HH practices and use of disinfectants may be helpful to increase compliance.

If the hands are not visibly contaminated, national and international guidelines on HH [9, 22] prefer hand disinfection with hand alcohol above applying soap and water. However, the majority of HCWs in our study group (63%) preferably used water and soap as method of HH. The presence and availability of ABHR for HH can significantly improve adherence to HH practice [34, 35]. Early quantitative studies of the effects of antiseptic handrubs established that alcohols, when used in concentrations present in alcoholbased handrubs, effectively reduce bacterial counts on hands [36, 37]. Typically, log reductions of the release of test bacteria from artificially contaminated hands average 3.5 log10 after a 30-s application [38]. We belief that the presence of ABHR in the working area and along the walking route together with effective educational programs about skin health, exact indications for using ABHR of water and soap will help HCWs to make this switch.

According to the literature, time is another reason for not practicing HH [34]_._ The median time of disinfecting hands was 8 s (range 6–11 s) for HCWs in our study. WHO has recommend hand disinfection time from 20 to 30 s [9], even this short time still seems to be too long in clinical practice. The actual time spent on a hand antisepsis action was reported to range between 5 and 24 s [39].

The researchers evaluated the efficacy of commercially available liquid ABHRs in vitro in an experiment in which 15 volunteers applied the hand rubs for 15 or 30 s. The ABHRs were “equal or even more effective” within 15 s vs. 30 s, with log reduction factors greater than 5 for *Staphylococcus aureus*, *Enterococcus hirae*, *Escherichia coli*, *Pseudomonas aeruginosa* and *Proteus mirabilis* and greater than 4 for *Candida albicans* when applied for 15 s, according to Kramer and colleagues [40]. In other study hand rubbing time of 15 s was not shown to be inferior to 30 s in reducing bacterial counts on hands and there was no gain in reducing bacterial counts from hand rubbing longer than 30 s [41]. Still, none of HCWs observed achieved even 15 s of hand-rubbing. Thus, concerns have been raised that application times that are too short may decrease ABHR antimicrobial efficacy [42]. We believe that a program to improve HH performance in general practice should focus on appropriate duration of HH performance by HCWs.

63% of HCWs in our sample wore hand and / or wrist jewelry during patient care. It has been found that skin under rings may be more heavily colonized with microorganisms than the rest of the hand, and that rings may also increase the risk of glove tears [9]. Wrist jewelry may prevent proper washing of the skin, and skin may not be dried properly following handwashing if wrist jewelry is present [9]. The consensus recommendation (national and international) is to strongly discourage the wearing of rings or other jewelry during health care.

Our study has some strengths and limitations. We observed 285 HH opportunities. Most of the observations were done among general practitioners and only a limited number of observations was done among NPs. In addition, our study population included only 30 professionals from 5 general practices. This precludes the statistical analysis of subgroups. Despite these limitations, we believe that our study is of value, as it is one of the few studies that evaluate HH compliance of HCWs in general practice. Future studies are needed on this topic with a bigger sample size to give us a better view on this topic. Also, we believe that when similar studies such as ours are carried out in future, other factors, including the impact of the coronavirus pandemic, which may greatly influence compliance to HH guidelines should be evaluated.

According to WHO Guidelines on HH in Healthcare recommendations [9], we measured HH compliance by direct observation. To reduce bias, one observer was trained and then validated. We minimized bias (the potential impact of the Hawthorne effect [43]) by not informing the HCWs about the exact purpose of the observations, although we cannot completely exclude that our observations resulted in an increased compliance with HH.

## Conclusion

HH compliance among health care providers in five Dutch general practices was found to be low, with no hand hygiene practice at all by GPs visiting patients at home. The WHO recommended switch from hand wash to ABHR was not implemented by the majority of health care workers. To improve HH compliance, besides promotional educational programs about skin health, indications and efficacy of HH practices, the presence of ABHR in the working area, along the walking route and availability of pocket bottles with ABHR may contribute to better hand hygiene performance in general practices.

## Data Availability

The dataset generated and analysed during the current study are available from the corresponding author on reasonable request.

## Abbreviations

*HH*: Hand hygiene
*HAI*: Healthcare-associated infections
*GPs*: General practitioners
*WHO*: World health organization
*HCWs*: Health care workers
*ABHR*: Alcohol-based hand rub
*PAs*: Practice assistants
NPs: Nurse practitioners
*IP*: Infection prevention
*WIP*: Dutch working group on infection prevention
*NHG*: Dutch general practitioners society
